# Supplementary figures and images for: Transcytosis subversion by M cell-to-enterocyte spread promotes Shigella flexneri and Listeria monocytogenes intracellular bacterial dissemination
Source: PLoS Pathog. 2020 Apr 13;16(4):e1008446. doi: 10.1371/journal.ppat.1008446 (PMC7179946; doi:10.1371/journal.ppat.1008446)

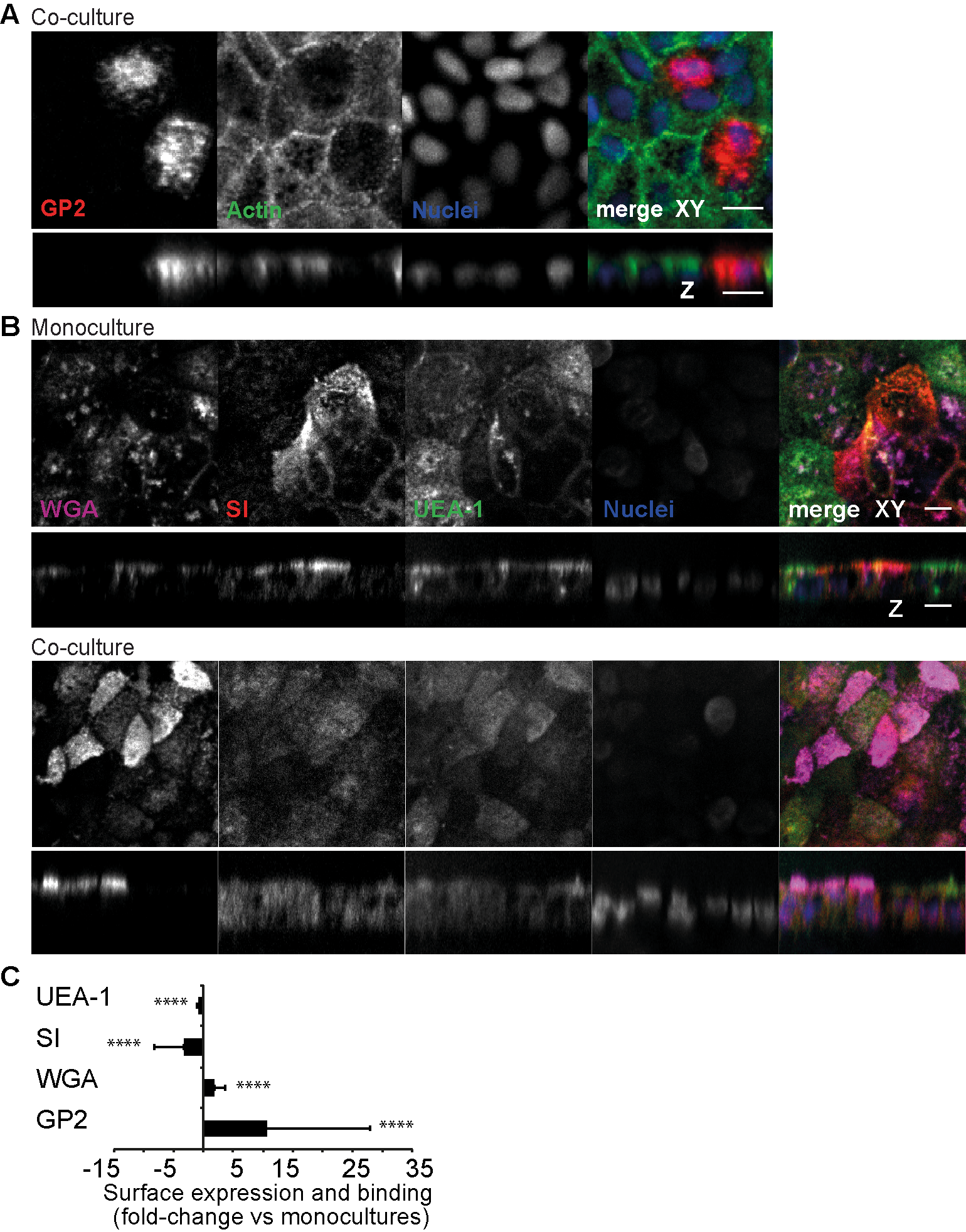

Supplement: S1 Fig — (A) Representative image of co-culture M cells identified by the apical expression of GP2 and the disorganized morphology of the apical brush border visible by actin staining (data from at least three independent experiments) Scale bar, 10 μm. (B) Representative images of mono- and co-culture apical binding of M cell specific WGA and enterocyte-specific UEA1; and apical expression of enterocyte-specific SI (data from at least four independent experiments) Scale bar, 10 μm. (C) Quantification of the fold-change difference in cells positive for M cell- and enterocyte expression markers (GP2/WGA and SI/ UEA-1, respectively) in co-cultures versus monocultures (GP2 n = 15, WGA n = 35, SI n = 25, UEA1 n = 20) Data is from at least four independent experiments. Data are mean ± s.d. (****p < 0.0001). (TIF) [file ppat.1008446.s001.tif]

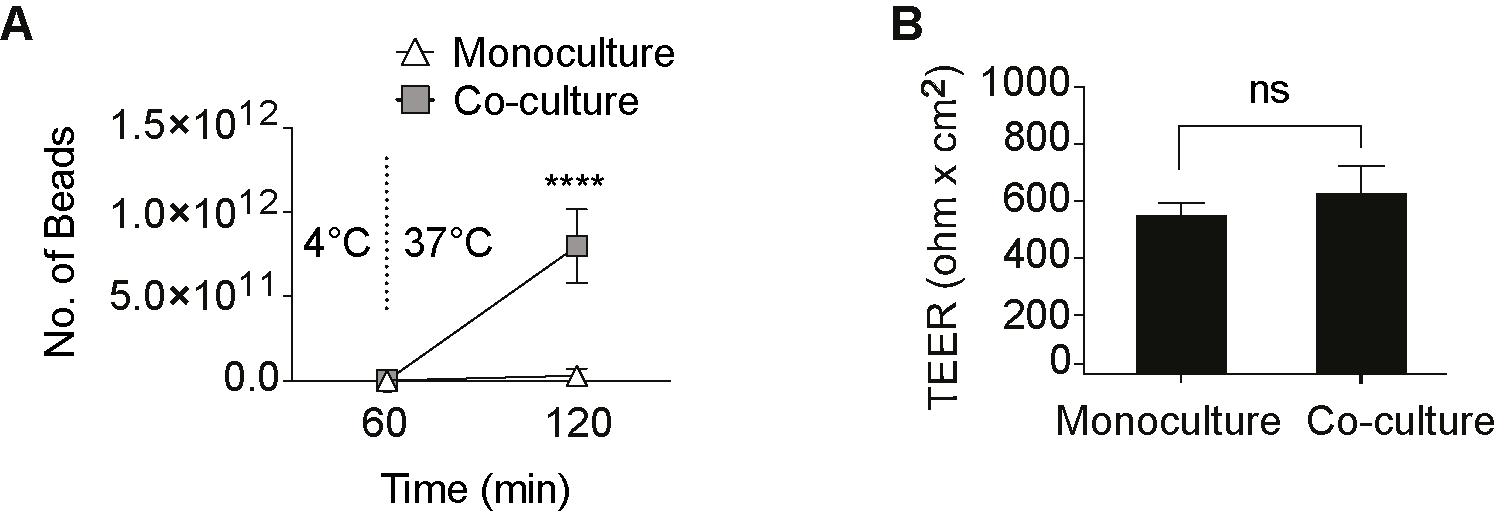

Supplement: S2 Fig — (A) Time-course of 20 nm carboxylated polystyrene bead translocation rates across mono- and co-cultures, with a temperature switch from 4°C to 37°C. Data is from four independent experiments. (B) Transepithelial electrical resistance (TEER) of control mono- and co-culture monolayers. Data are from five independent experiments. Data are mean ± s.d. (****p < 0.0001). (TIF) [file ppat.1008446.s002.tif]

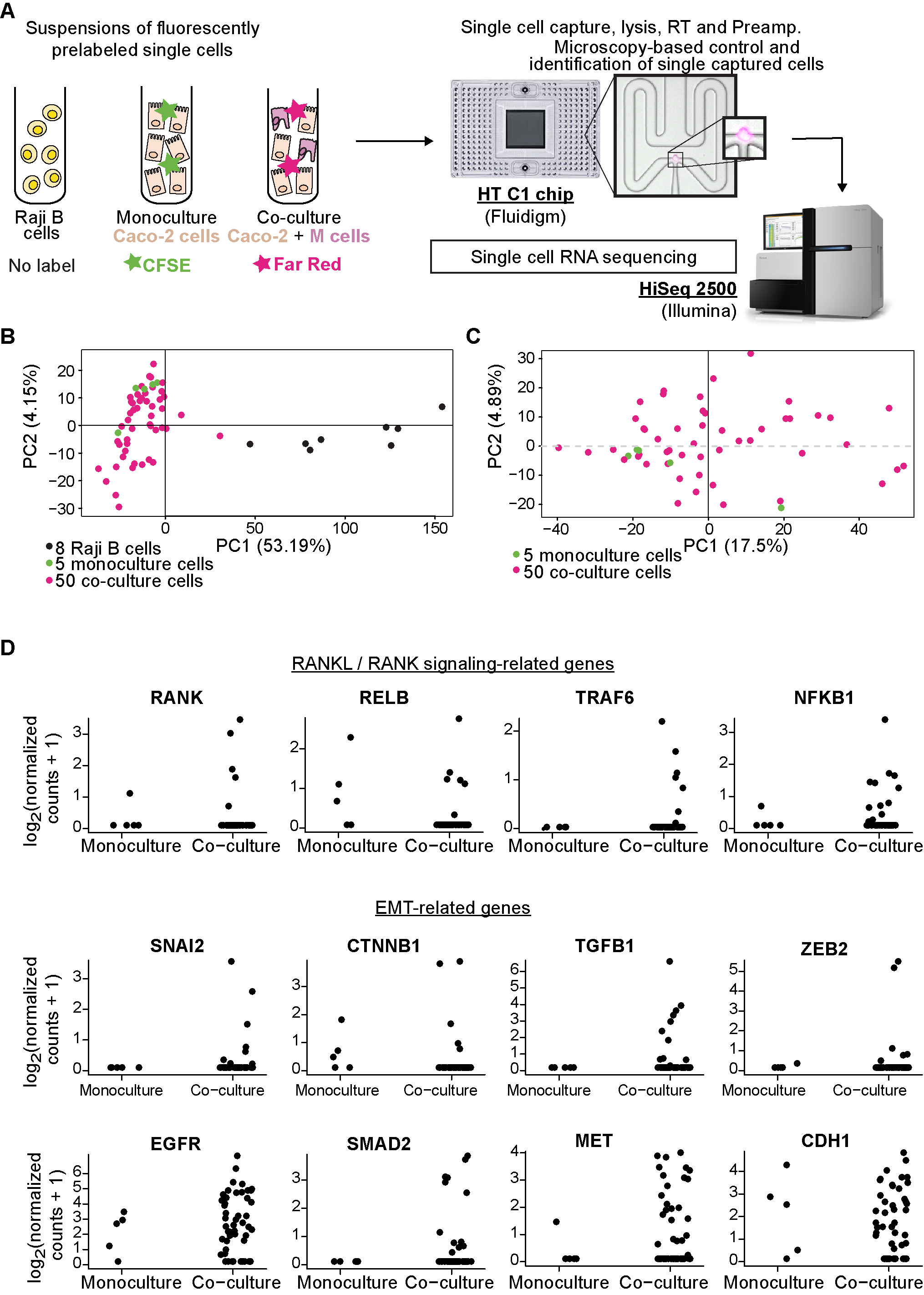

Supplement: S3 Fig — (A) Scheme of the workflow for isolation, identification and RNA sequencing of single co-culture M cells. Single cell suspensions of Raji B cells, CFSE-labeled monoculture Caco-2 cells and Far Red-labeled co-cultured Caco-2 and M cells are prepared and loaded as a mix onto a HT C1 chip (Fluidigm). The C1 system randomly captures single cells into individual capture sites. Each capture site is acquired at the fluorescence microscope to validate and identify single captured cells. The C1 chip is run for lysis, mRNA reverse transcription (RT) and pre-amplification of the cellular cDNA. Resulting single cell libraries of cDNA are prepared for HiSeq 2500 (Illumina) RNA sequencing. (B) Single cell transcriptomes of Raji B cells separate from control Caco-2 and co-culture cells along the first axis (PC1) of a principal component analysis projection (Raji B n = 8, Caco-2 n = 5, co-culture cells n = 50). (C) Single cell transcriptomes of co-culture cells and control Caco-2 cells, visualized by principal component analysis, suggesting the progressive acquisition of an M cell phenotype in co-culture Caco-2 cells (Caco-2 n = 5, co-culture cells n = 50). (D) Subsets of single co-culture cells express higher levels of genes of the RANKL / RANK M cell induction pathway, compared to control Caco-2 cells. Subsets of single co-culture cells express higher levels of genes of the epithelial-mesenchymal transition (EMT) pathway, compared to control Caco-2 cells (Caco-2 n = 5, co-culture cells n = 50). (TIF) [file ppat.1008446.s003.tif]

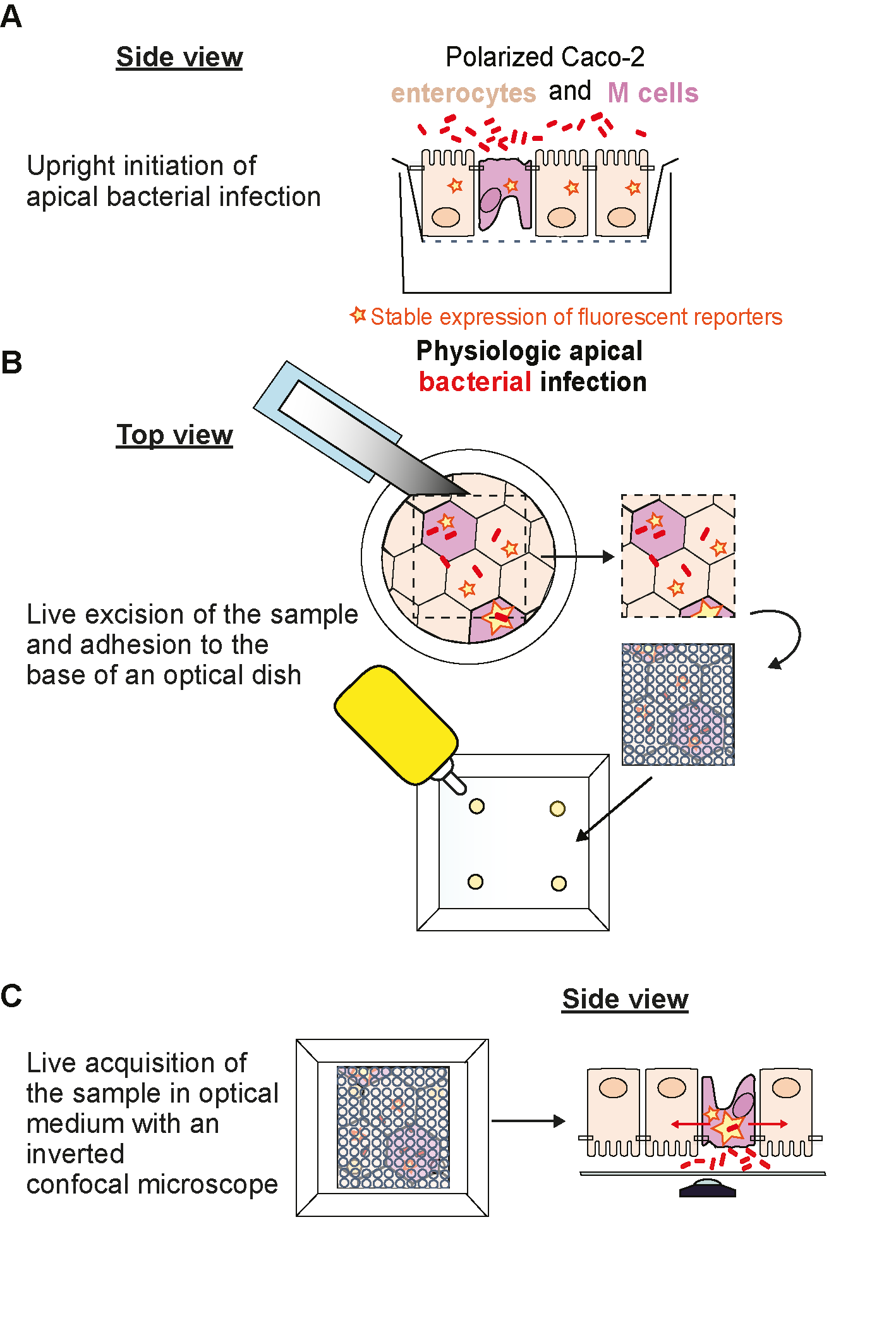

Supplement: S4 Fig — (A) Caco-2 co-culture M cells (magenta) and enterocytes (beige) are cultured on the membrane of a transwell. Stable fluorescent reporters and dyes are used to identify subcellular bacterial localizations, bacteria (red), label M cells and distinguish cellular membranes live. Apical initiation of bacterial infection is performed in an upright configuration to allow bacterial deposition on the epithelium by gravity. (B) Upon apical interaction of the bacteria with the epithelium, the transwell membrane is excised and adhered upside-down to the base of an optical dish with the apical side of the epithelium facing the bottom of the dish. (C) Optical infection medium is added to the dish and the sample is acquired up to 21 hours by time-lapse imaging using an inverted confocal microscope. (TIF) [file ppat.1008446.s004.tif]

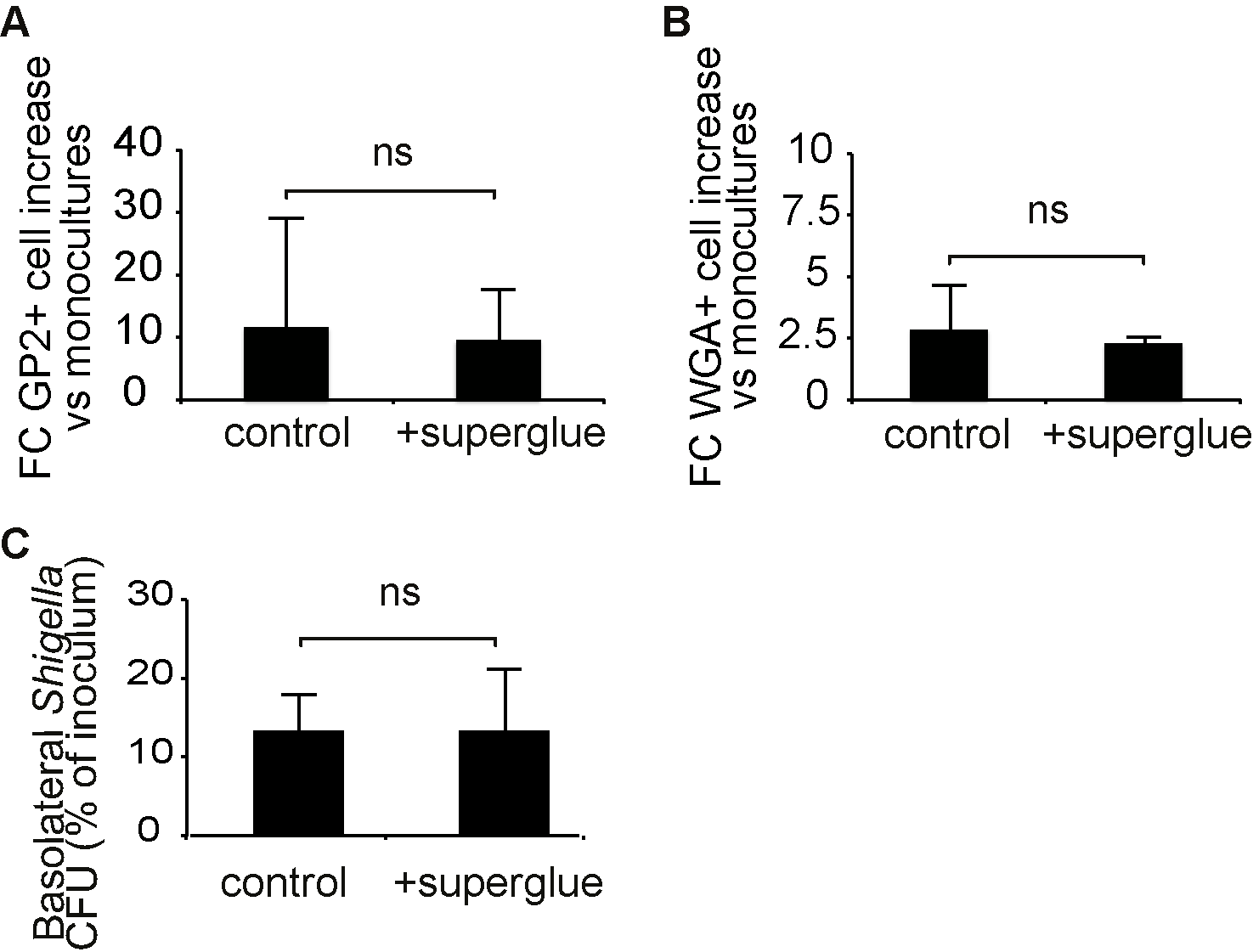

Supplement: S5 Fig — (A) A similar fold-change increase of GP2 positive M cells is observed in co-cultures excised and glued upside down for 1 hour, compared to non treated co-cultures, versus non treated monocultures (n = 3). (B) A similar fold-change increase of WGA positive cells is observed in co-cultures excised and glued upside down for 1 hour, compared to non treated co-cultures, versus non treated monocultures (n = 3). (C) Superglue treatment of co-cultures does not affect S. flexneri transcytosis (n = 3). (TIF) [file ppat.1008446.s005.tif]

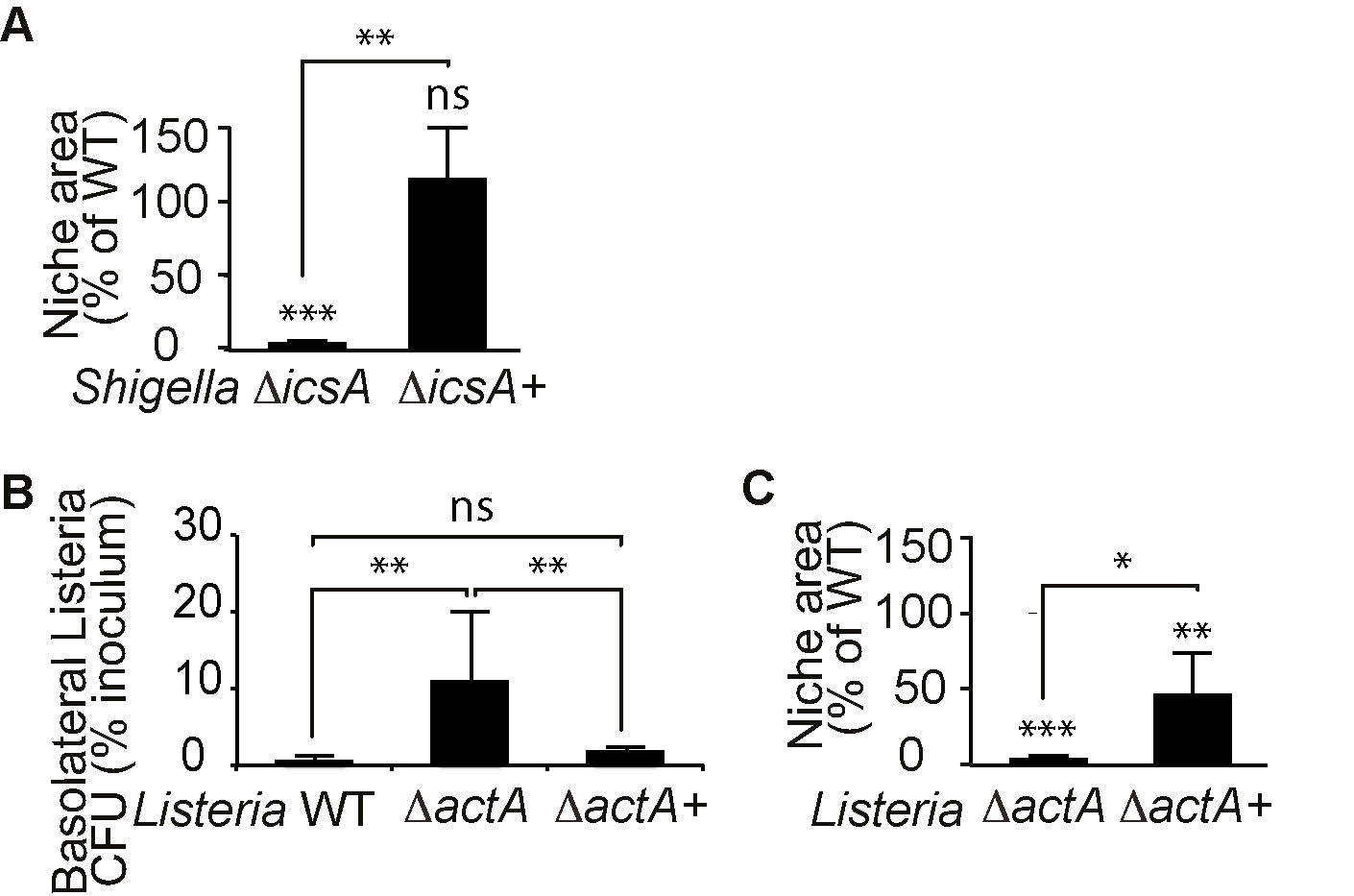

Supplement: S6 Fig — Complementation assays for S. flexneri ΔicsA and L. monocytogenes ΔactA phenotypes (A) IcsA complementation rescues the ability of S. flexneri ΔicsA to spread and form large infection niches at late time-points. The statistical significance of differences between conditions and WT were assessed by one sample t tests, and differences between conditions were assessed by a two-tailed unpaired t test (n = 3) (**p < 0.01, ***p < 0.001). (B) ActA complementation rescues the ability of L. monocytogenes ΔactA to subvert transcytosis through the co-cultures (n = 3) (**p < 0.01). (C) ActA complementation partially rescues the ability of L. monocytogenes ΔactA to spread and form large infection niches at late time-points, discussed in S1 Appendix. The statistical significance of differences between conditions and WT were assessed by one sample t tests, and differences between conditions were assessed by a two-tailed unpaired t test, (n = 3)(*p < 0.05, **p < 0.01, ***p < 0.001). (TIF) [file ppat.1008446.s006.tif]

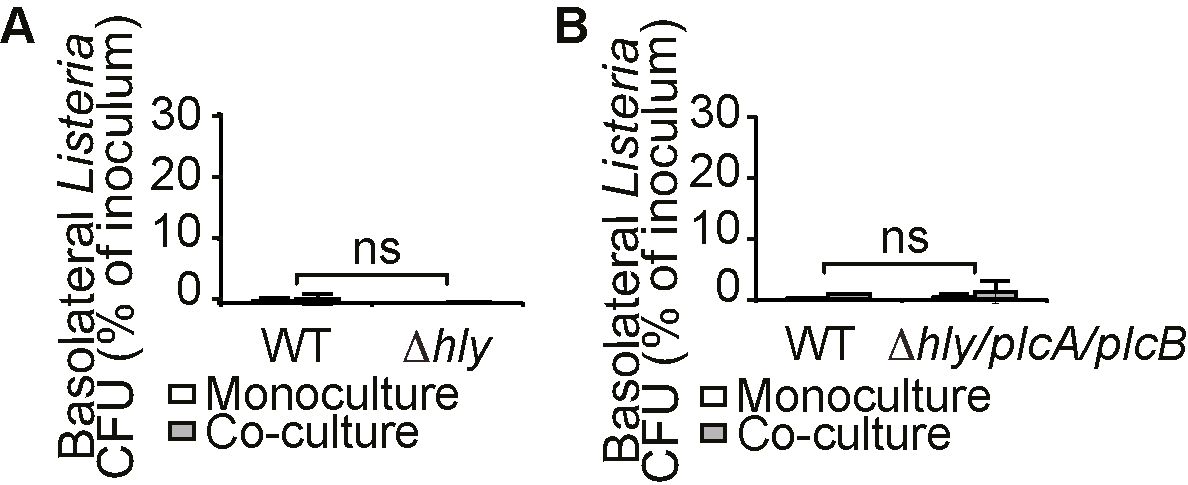

Supplement: S7 Fig — (A) The LLO mutant L. monocytogenes ΔactA does not transcytose through M cell containing co-cultures (n = 4). (B) The triple mutant L. monocytogenes Δhly ΔpIcA ΔpIcB does not transcytose through M cell containing co-cultures (n = 5). (TIF) [file ppat.1008446.s007.tif]
