# Supplementary material for: Transcytosis subversion by M cell-to-enterocyte spread promotes Shigella flexneri and Listeria monocytogenes intracellular bacterial dissemination
Source: PLoS Pathog. 2020 Apr 13;16(4):e1008446. doi: 10.1371/journal.ppat.1008446 (PMC7179946; doi:10.1371/journal.ppat.1008446)
Supplement: S1 Appendix — (DOCX) [file ppat.1008446.s010.docx]

**Supporting Appendix**

**Immunofluorescence-, transcytosis- and single-cell transcriptomic-based characterization of the human *in vitro* FAE model.**

The human co-culture M cell model was characterized following previously published procedures [1–5]. The analysis of the apical surface composition of the co-culture cells validated the presence of cells with typical apical M cell surface characteristics in our model (S1 Fig). Indeed, an increase of cells presenting a disorganized apical brush border, characteristic of M cells, was noted on average in 8% of the total co-culture epithelium. Furthermore, histochemical analysis of the FAE monolayers revealed similar proportions of cells expressing the M cell specific FimH receptor glycoprotein 2 (GP2) (10 ± 5%) and positive cells for wheat-germ agglutinin (WGA) binding, which binds with high affinity to the sialic-acid and N-acetylglucosamine residues present in M cells (10 ±8 %).

We quantified a 12-fold increase of cells expressing GP2 compared to monocultures (S1A and C Fig), and a 2-fold increase of cells labeled with the M cell marker WGA in the co-cultures (S1B and C Fig). Conversely, we measured a 0.5 fold and 0.6 fold decrease of cells expressing the enterocyte-specific differentiation markers Ulex Europaeus Agglutinin 1 (UEA-1), which binds to α-L-fucose and sucrase isomaltase (SI) in co-cultures respectively, compared to monocultures (S1B and C Fig); indicating the dedifferentiation of Caco-2 cells into M cells in the co-culture model.

Moreover, the capacity of the co-culture model to translocate material from the apical to the basolateral side was assessed following previously published procedures using 20 nm fluorescently labeled carboxylated polystyrene beads (S2 Fig, S2A Fig). No beads were detected in the basolateral compartments of mono- and co-cultures at 4°C. At 37°C however, a significant increase of bead translocation was observed in the co-cultures, suggesting an active transport via M cells. Monolayer barrier integrities were controlled by transepithelial electrical resistance (TEER) measurements showing similar TEERs (550 and 630 ohm.cm2) in monocultures and in co-cultures (S2B Fig). This confirmed the passage of the beads via M cell transcytosis through the co-cultures rather than the presence of disturbed cell-cell junctions, in agreement with previous reports.

We pursued the characterization of the human Caco-2 co-culture M cell model by single cell transcriptomic analysis, in a similar fashion to a recently published work focusing on murine M cells [6] (S3 Fig). Due to technical reasons, our analysis was restrained to a limited population of control Caco-2 enterocytes and Caco-2 M cell-containing co-culture cells, thus preventing the extraction of a clear M cell phenotype. In line with the lineage difference between lymphoid Raji B cells and epithelial Caco-2 enterocytes and M cells, we observed a clear separation of single control Raji B transcriptomes from control Caco-2 enterocyte and co-culture transcriptomes by principal component analysis (PCA) projection (S3B Fig). When we focused our PCA on control Caco-2 enterocytes and M cell containing co-cultures, we observed a higher dispersion of the co-culture cellular transcriptomes, suggesting the transformation of a subset of Caco-2 enterocytes towards the acquisition of an M cell transcriptional profile in the co-cultures (S3C Fig). In agreement with this hypothesis, we noted a higher expression of genes from the RANKL/RANK signaling pathway, involved in the transformation of M cells *in vivo*, in a subset of co-culture cells compared to control Caco-2 enterocytes (S3D Fig) [7,8]. We also observed the induction of epithelial-mesenchymal transition (EMT)-related genes in a subset of co-culture cells compared to control enterocytes, suggesting the involvement of this pathway in the transformation of differentiated Caco-2 enterocytes into M cells, as previously noted with bovine follicle-associated crypt (FAC)-derived Salmonella induced M cells (S3C Fig) [9,10].These results provided new insight into the involvement of relevant biological pathways for M cell differentiation in the co-culture model.

**Supporting References**

1. C. S, B. T, M. A, C. M, E. F, G. L, et al. Development of an advanced intestinal in vitro triple culture permeability model to study transport of nanoparticles. Mol Pharm. 2014;11: 808–818. doi:10.1021/mp400507g

2. Kerneis S, Bogdanova A, Kraehenbuhl J, Pringault E. Conversion by Peyer9s Patch Lymphocytes of Human Enterocytes into M Cells that ran sport Bacteria. 1996;277: 949–952. doi:10.1126/science.277.5328.949

3. Keita A V, Gullberg E, Ericson AC, Salim SY, Wallon C, Kald A, et al. Characterization of antigen and bacterial transport in the follicle-associated epithelium of human ileum. Lab Invest. 2006;86: 504–516. doi:3700397 [pii]\r10.1038/labinvest.3700397

4. Albac S, Schmitz A, Lopez-Alayon C, d’Enfert C, Sautour M, Ducreux A, et al. Candida albicans is able to use M cells as a portal of entry across the intestinal barrier in vitro. Cell Microbiol. 2016;18: 195–210. doi:10.1111/cmi.12495

5. Beloqui A, Brayden DJ, Artursson P, Préat V, des Rieux A. A human intestinal M-cell-like model for investigating particle, antigen and microorganism translocation. Nat Protoc. 2017;12: 1387–1399. doi:10.1038/nprot.2017.041

6. Haber AL, Biton M, Rogel N, Herbst RH, Shekhar K, Smillie C, et al. A single-cell survey of the small intestinal epithelium. Nature. 2017;551: 333–339. doi:10.1038/nature24489

7. Knoop KA, Kumar N, Butler BR, Sakthivel SK, Taylor RT, Nochi T, et al. RANKL Is Necessary and Sufficient to Initiate Development of Antigen-Sampling M Cells in the Intestinal Epithelium. J Immunol. 2009;183: 5738–5747. doi:10.4049/jimmunol.0901563

8. Walsh MC, Choi Y. Biology of the RANKL-RANK-OPG system in immunity, bone, and beyond. Front Immunol. 2014;5: 1–12. doi:10.3389/fimmu.2014.00511

9. Tahoun A, Mahajan S, Paxton E, Malterer G, Donaldson DS, Wang D, et al. Salmonella transforms follicle-associated epithelial cells into M cells to promote intestinal invasion. Cell Host Microbe. 2012;12: 645–656. doi:10.1016/j.chom.2012.10.009

10. Zhao M, Kong L, Liu Y, Qu H. DbEMT: An epithelial-mesenchymal transition associated gene resource. Sci Rep. 2015;5: 1–14. doi:10.1038/srep11459
