# Supplementary material for: Transcytosis subversion by M cell-to-enterocyte spread promotes Shigella flexneri and Listeria monocytogenes intracellular bacterial dissemination
Source: PLoS Pathog. 2020 Apr 13;16(4):e1008446. doi: 10.1371/journal.ppat.1008446 (PMC7179946; doi:10.1371/journal.ppat.1008446)
Supplement: Attachment — Submitted filename: Rebuttal_letter_2602.docx [file ppat.1008446.s011.docx]

**Comments to the journal after sending out the manuscript (points to be addressed before sending the manuscript back to the editor:**

We noticed that you used “data not shown” in the manuscript, we do not allow these references. The PLOS data access policy requires that all data be either published with the manuscript or made available in a publicly accessible database. Please amend the supplementary material to include the referenced data or remove the references. Please make a note of this change in your response to reviewers.

Ok, this has been amended.

We note that you have 4 figures and 0 figure legends. Please ensure that all figures are included and that each figure has a legend listed in the manuscript

Figure legends are now clearly portrayed in the manuscript

We note that we have 10 Supporting Information files and 9 Supporting Information legends. Please ensure that all Supporting Information files are included correctly and that each one has a legend listed in the manuscript

The Supporting Appendix (as 10^th^ supporting information) is now clearly cited in the text and marked in the manuscript.

We note that the following authors have not yet confirmed their authorship on this manuscript: Patricia Latour-Lambert, Caroline Proux.

We have reached out to the co-authors and hope you hear back from them. Of note, Caroline Proux has decided to travel independently (by boat) around the world. It is not easy to reach her these days.

**`Comments by the editor:**

1. **Relevance of the model. The physiological relevance of Shigella entry into M cells is controversial. There is little evidence of M-cell colonization by Shigella in humans or higher primates, where the primary site of colonization is the colon, which is largely devoid of M-cells. Rather the “widely accepted” M-cell colonization model has largely been derived from ligated ileal loop studies in rabbits, mice and guinea pigs. The authors should present a more balanced view of the literature in the Discussion and also tone down some of their “sweeping conclusions”. This applies not only to the text, but also the manuscript title.**

Considering the relevance of the model, we would like to comment on the scientific background that prompted us to study a human *in vitro* model of *Shigella* infections at the M cell enriched FAEs. It has been shown in rectal biopsy specimens of early cases of human Shigellosis patients that « aphtoid ulcers were present, particularly in the epithelium overlying lymphoid follicles » [1]. In the macaque, the initial site of *Shigella* invasion- determined using the non-motile Δ*icsA* Shigella mutant- was also reported to « essentially occur in the epithelium overlying lymphoid nodules » [2]. Together, these reports strongly suggest that the colonic follicle-associated epithelium (FAE) represents a major initiation point for Shigella infections.

The human colonic FAE is a monolayer of epithelium essentially composed of enterocytes and M cells [3]. It has been shown in rabbit ligated intestinal loop studies and *in vitro* studies based on differentiated Caco-2 cells (including our study here) that apical *Shigella* infection of enterocytes is very inefficient [4–6]. In contrast, *Shigella* has been shown to be rapidly uptaken by M cells in the rabbit ligated intestinal loop studies and in our study here; and FAE entry by the pathogen essentially occurred through M cells [5,7,8]. Taken together, these data strongly indicate that M cells represent a an important route for initiation of FAE infections. To our knowledge, while it has remained difficult to obtain direct evidence of *Shigella* interactions with M cells in human patients and macaques, there has been no clear evidence until now for initial targeting of other cellular routes in these models either.

Regarding the presence of M cells in the large intestine, intestinal lymphoid follicles (ILFs) are estimated at around 30,000 in humans (while Peyer’ patches culminate at 250 in the mid-teens), with high densities in the human distal colon- playing a crucial role for intestinal homeostasis and containment of the microbiota [3,9–11]. Moreover, the presence of M cells at the FAE overlying human colonic ILFs has been thoroughly established [3,12–14].

With regards to all these points, we believe that our model was chosen based on pertinent grounds and that the canonical model of M cell targeting by *Shigella* at the FAE, during the onset of human infections, is supported by consistent evidence.

Nevertheless, we understand that these different models may have limitations, and we agree to present our work with a more balanced view of the literature. To do this we referred to more recent data published on *Shigella* invasion of intestinal crypts in the Introduction and more generally on alternative intestinal invasion routes by intracellular pathogens in the second paragraph of the Discussion. We appreciate your comment that we should tone down our sweeping conclusions in the Discussion and manuscript title. To this end, we did changes throughout the Discussion to keep our statements more guarded and changed the manuscript title from « *Shigella flexneri* and *Listeria monocytogenes* subvert microfold cell immunological sampling by direct actin-based M cell-to-Enterocyte dissemination » to « Transcytosis subversion by M cell-to-enterocyte spread promotes *Shigella flexneri* and *Listeria monocytogenes* intracellular bacterial dissemination ».

2. **Complementation of Shigella mutants (Major point 1, reviewer 2).**

We appreciate the added value of including complementation controls for the *Shigella* IcsA mutant and have performed revisions experiments with this control. Moreover, we also performed extensive complementation controls for the *Listeria* ActA mutant, which have been included in the revised manuscript (Fig S6).

3. **Statistical analysis (Reviewer 3)**

We have modified the statistical analysis and updated the results in the manuscript (multiple figures, Supplementary figures, legends and Materials and Methods).

4. **The validity of gluing monolayers before live cell imaging (Reviewer 3)**

We have performed controls regarding the impact of gluing monolayers in our system and have included them in the updated version of the manuscript (Fig S5).

**Comments by the reviewers:**

**Reviewer #1: In this paper, Eninga and colleagues designed a tissue culture systems to investigate the role of M cells in Shigella flexneri infection and dissemination. In this model, S. flexneri develops a bimodal lifestyle within M cells that involves (1) rapid transcytosis or (2) vacuole escape followed by actin motility-based motility and spread to adjacent enterocytes. The authors argue that their results provide a potential mechanism for the intracellular pathogen ability to evade adaptive immunity. Although the experiments are appropriately conducted, it is unclear why the authors launched such a research program to begin with. The model of S. flexneri infection involving M cells originated from studies conducted in the small intestine, which is not the site of S. flexneri infection in humans. Moreover, recent studies strongly suggest that in the colon, which lacks M cells, Shigella directly infect epithelial cells. So this reviewer appreciate the technical tour de force displayed here, but unfortunately the biological significance of the project and the approach is groundless.**

We would like to thank Reviewer#1 for taking the time to evaluate our manuscript and for appreciating the technical aspect of our work, although it is very unfortunate to us that we were unable to convince this reviewer of the relevance of our endeavor. In our response to the first point of the general review recommendations by the editor (see the text above) we have commented the scientific background upon which we based the rationale of our study. Also, we decided to rephrase numerous expressions in our manuscript to make it clearer that each experimental model has limitations. We hope we can convince Reviewer#1 that M cell research in the context of enteric and colonic infections, *Shigella* included, should not be closed. Indeed we believe that this is an important area of research for the understanding of mucosal diseases, which would benefit from more attention from the scientific community.

**Reviewer #2: M cells have been demonstrated in the past to represent a major entry port of important human enteropathogens such as Shigella and Listeria. In addition, lateral cell-to-cell spread in a monolayer of epithelial cells has been demonstrated for both Shigella and Listeria. The rapid and efficient translocation of bacteria by M cells, however, prompted the idea, that M cells provide the port of entry and that both bacterial pathogens subsequently re-enter the epithelial layer from the basolateral side to spread cell-to-cell.
Instead, Rey et al. now show that M cells provide both, (i) port of entry to allow the translocation and (ii) the initial focus of lateral epithelial spread. They demonstrate that a fraction of intracellular bacteria in M cells lyse the trafficking endosomal compartment, acquire cytosolic motility spread from the M cell to the neighboring enterocytes generating a focus of infected cells.
Although the mechanisms involved are not novel per se, the careful time-lapse resolution and differentiation between basolateral re-infection and M-cell-derived lateral cell-to-cell spread generate a significant conceptual advancement and may explain previous immunological findings. The study is well-performed using state of the art tools (reporter constructs, high end microscopy, life imaging) and is certainly of interest for a broader readership in the field of infection research and mucosal immunology. The reviewer feels that the results of the study do perfectly justify publication in PLoS Pathogens.**

We would like to thank Reviewer#2, for the time spent evaluating our manuscript, and would like to express our gratitude for the positive feedback on our experimental approach and scientific results.

**Reviewer #2: Major points :
1. If possible also provide the results of complemented bacterial strains (Fig. 2b, Fig. 3b, Fig. 4a).**

We have conducted complementation experiments and have included the results in the supplementary data (Fig S6), as different sets of experiments were used in the main figures. For *Shigella*, we used the previously published SC560 Δ*icsA* and SC561 Δ*icsA* + complemented strains to verify the contribution of IcsA to the area of *Shigella* epithelial infection niches at late timepoints (Fig S6A, related to Fig. 3b, [15]). We observed a complete phenotypic rescue of infection niche areas after IcsA complementation. These results confirmed that the observed reduction of niche size with the IcsA mutant is specifically due to the inactivation of the *icsA* gene (Fig. 3b). Of note, we did not perform the complementation experiment for the mxiD mutant (Fig. 2b) as the role of mxiD for *Shigella* invasion has been well documented, including in the context of M cell entry, and this aspect of the work isn’t new [7,8,16]. Moreover, as we established that IcsA does not play a role in transcytosis, we did not perform an IcsA complementation for this experiment either (Fig. 2b).

With *Listeria,* we used the BUG 2140 EGD Δ*actA* strain and transformed this strain with the plasmid p*actA3* containing the *actA* structural gene and immediate upstream promoter, thus generating the complemented BUG 2140 EGD Δ*actA* + strain [17]. We observed a complete rescue of M cell transcytosis subversion after ActA complementation (Fig S6B, related to Fig. 4a).

In addition, we performed an ActA complementation experiment for intercellular Listeria spread at late timepoints (Fig S6C, related to Fig. 4H). Here, we only observed a partial rescue (50%) of the spreading area of Listeria infection in the monolayers after complementation. We believe that this partial phenotype rescue is due to the absence of the *mpl* promoter further upstream of *actA* in our complemented mutant, which has been reported to generate a *mpl-actA-pIcB* transcript. It has previously been reported in L2 cells that the deletion of the *mpl* promoter results in Listeria plaques that are 60% the size of WT [18]. Here we observe a slightly higher decrease, which could be explained by the potential involvement of ActA in specific M-cellular processes, such as internalization through an independent pathway. Therefore, the incomplete expression of ActA in the complemented strain may have an increased impact on the effective spreading of the bacterium in our model compared to L2 cells. We also noted a decreased average length of the comet tails produced with the complemented strain compared to wild-type in non-polarized Caco-2 cells, however this is difficult to quantify. This notion provides additional hints about the reduced potency of this strain for intercellular motility.

**2. Are there any preferences in the evasion-promoting activity of the M cell endosomal compartment as compared to the endosomal compartment of absorptive enterocytes, e.g. in respect of the lytic effectors of Listeria (Hly, PlcA or PlcB)**

With respect to M cell vacuolar rupture by *Shigella* (occuring at 74 $\pm$ 33 min p.i.), we observed a delay compared to Caco-2 enterocytes by time-lapse microscopy (22 $\pm$ 1 min p.i.). As discussed in the manuscript, our hypothesis for this delay is that owing to its unique architecture designed for antigen sampling, the endosomal M cell compartment may be more difficult to subvert by *Shigella* for vacuolar rupture.

For *Listeria*, we were unable to follow the dynamics of vacuolar rupture by time-lapse imaging and therefore we have no data regarding timing differences of this phenomenon between M cells and enterocytes. In enterocytes it is known that LLO, encoded by the gene *hly,* is a major virulence factor involved in vacuolar rupture. We tested the role of LLO in promoting M cell transcytosis subversion through vacuolar escape in the co-cultures. Surprisingly we observed no increased transcytosis with the Δ*hly* mutant compared to WT, indicating that LLO does not play a role in transcytosis subversion by *Listeria* (Fig S7A).

We then tested a triple *Δhly,* Δ*pIcA*, Δ*pIcB* vacuolar mutant form of the EGD-e strain prfA* to rule out the involvement of pIcA and pIcB in vacuolar rupture and transcytosis subversion. Again, we observed no impact on M cell transcytosis compared to WT (Fig S7B).

Taken together, it appears that the canonical *Listeria* effectors of vacuolar rupture LLO, pIcA and pIcB do not play a role in transcytosis subversion by the pathogen in M cells. We included this data in the manuscript and discussed this. We hypothesize that *Listeria* employs an alternate ActA-dependent internalization route in these cells which may rely on other factors for vacuolar escape. Interestingly, we observed a slight trend for ActA-dependent perturbation of antigen trafficking during M cell infection, which may suggest the solicitation of the endomembrane compartment during transcytosis subversion (see also Reviewer#3 point 2 response below).

**Reviewer #2: Minor points:**

**1. Add space: Line 87.**

Done

**2. Check italic style for in vitro/in vivo and mutans.**

Done

**3. Line 155, the infection time starts from 40 min.**

Regarding this comment, we are sorry if the text related to the figure was unclear. In Fig2C we showed a representative image of bacterial adhesion, which in this occurrence was observed at 40 min p.i., although events of apical *Shigella* adhesion to M cells were typically observed from the beginning of our imaging sessions at around 20 min p.i.. We have clarified this in the text:

« Instead, we observed extended events of apical bacterial adhesion to WGA-positive M cells after the occurrence of the majority of transcytosis events, starting from 20 min p.i. (Fig 2C, S1 Movie) » was changed to « Instead, we observed extended events of apical bacterial adhesion to WGA-positive M cells after the occurrence of the majority of transcytosis events, starting from the beginning of our imaging acquisition sessions at around 20 min p.i. (Fig 2C, S1 Movie). »

**4. "Fig 4C,D" in line 198 should be moved to line 196.**

Done

5. **Line 331, µ not Italic.**

Done

**6. Line 345, 5µm instead of 5um.**

Done

**7. Line 493, add bracket.**

Done

**Reviewer #3: This manuscript by Rey et al. investigates the mechanisms of initial infection of follicle-associate epithelia (FAE) by invasive bacterial pathogens using an in vitro microfold (M) cell model. The authors describe the validation of the previously established human M cell model and determine its appropriateness to model bacterial infection. Using a combination of transcytosis measurements and confocal time-lapse microscopy, infecting Shigella flexneri are described as having two fates: either being rapidly transcytosed or lysing the M cell vacuole in a delayed manner. Use of different bacterial strains uncovers a role for actin-based motility in the lateral dissemination into neighboring enterocytes by the latter population. Furthermore, similar experiments with Listeria monocytogenes lead the authors to propose that this evasion of M cell transcytosis by actin-based motility phenomenon is not specific to Shigella.

The results generally support the overall conclusions made by the authors. However, the authors tend to make sweeping conclusions in places where the data are lacking; although the results here are likely to support the general conclusions, the story would be more convincing if the authors were more circumspect in their interpretation of the data in these places.**

In response to Reviewer#3’s general comments, we would like to thank him or her for carefully evaluating our manuscript and providing a constructive feedback regarding the results and their presentation to make our study more convincing. We hope we were able to clarify the areas of the study that were unclear and to express our conclusions with more circumspection in the revised document. We have also changed the title.

**Reviewer #3: Minor points**

1. **Live cell imaging: the technique employed was to allow bacteria to enter cells for 30 minutes, then to glue the monolayer in an inverted orientation to a mount and image live. More controls are needed to test whether critical M cell functions are not altered by using super glue to adhere the apical surface to a fixed polymer surface. Does transcytosis of beads (and S. flexneri) continue to occur at the rates that it occurs in non-inverted/glued co-cultured cells? Do the M cells continue to produce WGA ligand(s) and GP2?**

To address the requirement of more controls regarding the impact of superglue and inversion of excised co-culture monolayers for time-lapse microscopy on M cell biology, we performed an imaging-based analysis of M cell markers on inverted/glued co-culture cells and performed *Shigella* transcytosis experiments in presence of superglue.

Regarding the preserved ability of the co-cultures to transcytose beads, we did not excise the samples as this would create a continuous apical/basal compartment and prevent the discrimination of transcytosed basolateral beads from the apical inoculum. Instead, we performed a conventional upright transcytosis assay but in presence of superglue drops, as employed for gluing of the inverted monolayer during time-lapse microscopy. We observed no impact of the superglue on *Shigella* transcytosis in 3 independent experiments (Fig S5C).

Moreover, compared to the previous control data presented in Fig. S1, we observed no significant difference in numbers of GP2 and WGA positive cells after 1h of treatment in inverted/glued configuration compared to monocultures treated in normal upright configuration. This experiment was performed independently 3 times (Fig S5A-B).

These results further consolidated our observation that the inversion and gluing of differentiated monolayers is an innocuous, efficient and easily accessible way to obtain high spatiotemporally resolved images in polarized tissues. Indeed, we were able to observe preserved dynamics of intercellular Shigella infection spread during our imaging sessions, as well as preserved morphologies of the epithelial cells throughout the timelapses. Of note, we used cyanoacrylate glue (“superglue”) following a recommendation for maintaining live cell or tissue slices during imaging from a Cold Spring Harbor protocol [19,20]. We commented on these results in the Results section.

**2. The proposition that delay for T3SS-dependent hijacking of vacuole trafficking in M cells due to the inherent high levels of endomembrane compartment trafficking in these cells is interesting. The authors could test the impact of bacterial factors on the rate of transcytosis in M cells by doing the bead transcytosis experiments in concert with infection with bacteria wild type strains and their respective actin-based motility defective mutants. If IcsA and ActA are involved in the dysregulation of transcytosis, the number of beads recovered from co-infection with the WT strains ought to be decreased compared with those recovered from co-infection with the icsA and actA strains.**

We would like to thank Reviewer#3 for suggesting this interesting experiment, building upon our thoughts presented in our Discussion. We performed the suggested experiment by measuring the translocation rates of beads, which were added 15 min after adding the bacterial to the monolayers at room temperature and before initiation of the infection at 37°C. We compared the translocation rates of the beads at 1h p.i. with Shigella and Listeria WT and their non-motile *ΔicsA* and *ΔactA* counterparts. These experiments were performed 5 independent times for *Shigella* and 3 independent times for *Listeria*.

For *Shigella*, we observed a 1.4 ± 0.7 fold change increase of beads translocation in Δ*icsA* treated samples compared to WT.

With *Listeria*, we observed a 1.2 ± 0.1 fold change increase of beads translocation in Δ*actA* treated samples, compared to WT*.*

Although these results were not statistically significant, they may suggest a trend for effector-driven perturbation of M cell antigen trafficking during intracellular pathogen infection, possibly to promote vacuolar rupture. For *Listeria*, this could complement the results presented higher, which exclude a role for the canonical vacuolar rupture effectors LLO, pIcA and pIcB in the subversion of M cell transcytosis. It would be interesting to assess further the impact of intracellular pathogen virulence effectors on M cell antigen sampling, by testing more conditions and different virulence effectors in the future. We think that this would require an independent study.

**3. Fig 1: The figure legend should provide more of the relevant details. Panels B and D, define which cells are in “monoculture” and indicate that they are polarized. Panel C, define what the “control” is.**

Fold-change increase in bead translocation vs WT

|  | Average |
| --- | --- |
| Listeria ΔActA | 1.19644 |

Fold-change increase in bead translocation vs WT

|  | Average |
| --- | --- |
| Shigella ΔIcsA | 1.39873 |

Thank you for these comments, we have amended the text in the figure legend accordingly.

**4. Fig. 2. In panel C, are all images from the same field? If so, why does the shape of the outline change at 80 and 110 minutes?**

Yes, all the images are from the same XY field, but at different Z-planes and time-points, to capture the best view of the different events occurring at different depths within the M cell (internalization is apical, vacuolar rupture and bacterial replication are more basal in the cell). We captured the entire Z-stack at each timepoint and have observed that the outlines of polarized cells are different throughout the Z-stacks and are dynamic and can therefore change over time.

We have clarified this in the figure legend.

**5. How were the perimeters of the individual cells (the dashed lines) determined?**

The perimeters of the individual cells were manually delineated after visual assessment of each fluorescent channel in the XY, in the Z and over time (the FM-464 membrane dye provides a peripheral marker by staining the cellular plasma membrane, galectin 3-eGFP provides a cytosolic marker which visually complements the delineation of the periphery of the cell when vacuolar rupture isn’t occurring, villin-eYFP is enriched at the cortex of the cell, thus providing another visual marker of the cellular periphery)**.**

We have clarified this in the Materials and Methods section.

6. **Define WGA, GP2, UEA1, SI.**

Thank you for this comment, we have defined these terms in the S1 Appendix and in the Materials and Methods. **7. Lines 116-118: Although the authors avoided some of the tools that investigators use to enhance infection, it is not true that they avoided all of them, as according to the Methods, the infecting strain appears to produce the E. coli Afa-I adhesin; this should be mentioned here and in the 3rd paragraph of the Discussion (lines 238-240).**

We are sorry if the text was unclear in the Methods here, we only employed an infecting strain producing the E. coli Afa-I adhesin for the time-lapse experiments, reducing the MOI even further by half, to avoid their detachment from the apical side of the monolayers upon excision and inversion for imaging (we clarified this in the Methods, and in the Results section). As we shortened our discussion of this part in the revised Discussion (following your point below, 12.), we did not comment on this further in this section.

**8. Lines 132-133 (Fig. 1E): “The time-lapses confirmed the exclusive apical targeting of M cells by S. flexneri…” The data presented in Fig. 1E show a representative image of S. flexneri infecting an M cell. They do not show any quantification that might support the conclusion that infection was (1) “exclusively apical” or (2) “exclusively of M cells.” Either the authors need to be more circumspect in their conclusions or show the data that support these conclusions.**

We performed apical infection experiments in monocultures of differentiated Caco-2 cells and only observed infection niches occurring in co-cultures of differentiated Caco-2 and M cells (Fig 1D), in conditions of preserved barrier integrity (Fig 1C); suggesting the exclusive apical targeting of M cells for initiation of *Shigella* infection in our experimental setup (this was performed in more than 5 independent experiments). Moreover, as mentioned in the Results section, we only observed infection events occurring in cells that were positive for the M cell marker WGA in our time-lapses, after apical challenge of the monolayers (this was observed in more than 3 independent experiments). We did not observe events of basolateral infections in this assay during initiation of the infection. As no events of apical or basolateral initiation of infection in enterocytes were observed in the assays mentioned higher, we have no relative data to support quantifications here.

Furthermore, in initial MOI tests we noted an inverse correlation of Shigella transcytosis with an absence of bacterial translocation at higher MOIs (up to 300 bacteria per cell), as well as an absence of infection niches at late time-points in fixed co-cultures. We noted therefore that uptake of *Shigella* by M cells is not linear to the number of apical bacteria and that the initiation of infection niches in co-cultures of Caco-2 M cells and enterocytes seems to only occur at low MOIs- making it difficult to address your question quantitatively.

**9. Statistical methods: The authors state (lines 611-613) that differences between experimental conditions were determined by t tests. Whereas t tests are appropriate for experiments that consist of only two experimental conditions, for experiments in which three or more conditions were used, analysis of the data by ANOVA is more appropriate and should be used here. In addition, where two conditions are present, if the data are transformed (e.g., to percentages), the t test is also generally not appropriate. Moreover, where the t test is appropriate, the assumption of a Gaussian distribution appears to have not been assessed.**

Thank you for these comments, we have updated the statistical analysis in the manuscript (all the figures were reanalyzed and their presentation was changed according to the updated statistical analysis presented in the Materials and methods).

**10. In Fig. 4D, the presentation of the data is statistically incorrect: since the sum of the two bars is necessarily 100%, the two bars should not be directly compared by statistics. An appropriate presentation of the data would be to have a single bar that totaled 100%, and within that bar to indicate the proportion infected cells that are enterocytes and the proportion that are M cells. Moreover, these data are really only relevant if compared to a quantification of the percentage of the cells in the analyzed monolayers that were M cells (and the percentage that were enterocytes); these numbers are not clearly presented anywhere in the manuscript.**

Thank you for these comments, we have changed the presentation of the data for Fig 4D. We have clarified the numbers of M cells in the Supporting Appendix and have included these numbers in the Results section of the manuscript.

**11. Regarding L. monocytogenes, the suggestion in the Figure 4 legend title and in the Results sub-heading is that it behaves similarly to S. flexneri, whereas the results indicate that it behaves differently, in that transcytosis is detectable only in the absence of actin-based motility. These headings are therefore a bit misleading; the authors should consider rewording them to better reflect the findings.**

Thank you, we have changed these titles to reflect more the differences between the lifestyles of both pathogens during M cell infection.

**12. As currently written, the Discussion is largely a regurgitation of the results. Therefore, it could be shortened substantially. On the other hand, how the antigen-sampling function of M cells might relate (or not) to its use by pathogens would be a good topic on which to expand the Discussion.**
Thank you for these comments, we have shortened redundancies in the second paragraph of the Discussion to and have expanded on the antigen-sampling function of M cells in the third section of the Discussion.

**13. Whereas the authors claim that the system is physiological, Fig. S1C indicates that the co-culture leads to nearly 100% of cells producing the M cell markers WGA ligand(s) and GP2 and approximately 50% of cells producing the epithelial cell markers UEA1 and SI. There are two issues here that should be addressed: (1) in the human colon, M cells are the minority of cells (nowhere near 100% of cells), and (2) what type of cells are the approximately 50% in this system that produce both M cell markers and epithelial cell markers?**

We are sorry if the results are unclear here. In Fig S1C the decrease of positive cells for M cell markers in monocultures is presented, relative in percentage to the total number of positive cells in the co-cultures. To be clear, only about 10% of the co-culture cells are positive for the M cell markers, reminiscent of the proportion of M cells found at the FAE in the human colon.

Conversely, the decrease of positive cells for enterocyte-specific markers in the co-cultures is presented, relative in percentage to the total number of positive cells in the monocultures.

To clarify the presentation of the data, we replaced this figure with a bar chart of fold-changes in numbers of positive cells for M cell (GP2 and WGA) and enterocyte (SI and UEA-1) markers in co-cultures versus monocultures (Fig S1C).

**14. Fig. S3: Transcriptional data show that compared with monocultured cells, subsets of co-cultured cells express higher levels of genes of the RANKL/RANK M cell pathway and the epithelial-mesenchymal transition pathway. With cluster or other analysis, is it the same subset of cells that are expressing higher levels of each of the transcripts that are shown here as a separate graph or are these distinct subsets?**

In this analysis, we noted that these genes are not upregulated in distinctive subsets of cells, possibly reflecting contrasting stages of maturation among the co-culture cells. **15. In Appendix S1, the authors state that the transcriptomics analysis of the co-cultures offers “new insights into the involvement of relevant biological pathways for M cell differentiation”. It seems to this reviewer that it is the reverse, that the transcriptomes were analyzed to assess whether expression profiles characteristic of M cells were present in the co-culture model, and thus few new insights were offered?**
We are sorry if our statement was unclear. As M cells are rare and interspersed within the FAE, we performed a transcriptomic analysis of individual FAE cells, to extract the specific transcriptional profile of M cells. This provides new insights into the Caco-2 M cell model, as previous transcriptomic studies with this system were performed in whole populations of co-culture cells, diluting distinctive features of M cell transcriptomes within the dominant population of enterocytes[21]. Our results show the upregulation of the RANKL/RANK M cell pathway, which has been shown to be involved in other models of M cell differentiation, but not in this *in vitro* system so far. Moreover, the upregulation of the EMT pathway has not been reported previously in this model.

**16. In certain places, the wording is unclear: lines 153-159 and 489-493.**

Thank you for these comments, we clarified the text for the first passage. With regards to the second passage, we do not know how to write this better. We describe the change of the buffer with different gentamicin conditions and also the addition of FBS, we think that this is clear.

**17. Fig 4C: It would be informative to add an image of infection with the actA mutant.**

Thank you for this suggestion, we performed this experiment, but as this strain is transcytosed by the M cells, there were very few obvious infection events at this early timepoint in the co-cultures and the data did not seem informative. Therefore we decided not to include this image in the manuscript. **18. References: reference 29 appears to be incorrect, with little mention of Listeria in the context of translocation across the FAE. Several references have author duplications (e.g. references 20, 26, …).**

Thank you for this comment. We have changed and corrected the references.

**References**

1.	Mathan MM, Mathan VI. Morphology of rectal mucosa of patients with shigellosis. Rev Infect Dis. 1991;13: S314–S318. doi:10.1093/clinids/13.Supplement_4.S314

2.	Sansonetti P, Arondel J, Fontaine A, D’Hauteville H, Bernardini ML. OmpB (osmo-regulation) and icsA (cell-to-cell spread) mutants of Shigella flexneri: vaccine candidates and probes to study the pathogenesis of shigellosis. Vaccine. 1991;9: 416–422. doi:10.1016/0264-410X(91)90128-S

3.	O’LEARY AD, SWEENEY EC. Lymphoglandular complexes of the colon: structure and distribution. Histopathology. 1986;10: 267–283. doi:10.1111/j.1365-2559.1986.tb02481.x

4.	Mounier J, Vasselon T, Hellio R, Lesourd M, Sansonetti PJ. Shigella flexneri enters human colonic Caco-2 epithelial cells through the basolateral pole. Infect Immun. 1992;60: 237–48. Available: http://www.ncbi.nlm.nih.gov/pubmed/1729185

5.	Perdomo OJ. Acute inflammation causes epithelial invasion and mucosal destruction in experimental shigellosis. J Exp Med. 1994;180: 1307–1319. doi:10.1084/jem.180.4.1307

6.	Romero S, Grompone G, Carayol N, Mounier J, Guadagnini S, Prevost M-C, et al. ATP-Mediated Erk1/2 Activation Stimulates Bacterial Capture by Filopodia, which Precedes Shigella Invasion of Epithelial Cells. Cell Host Microbe. 2011;9: 508–519. doi:10.1016/j.chom.2011.05.005

7.	Wassef JS, Keren DF, Mailloux JL. Role of M cells in initial antigen uptake and in ulcer formation in the rabbit intestinal loop model of shigellosis. Infect Immun. 1989;57: 858–863.

8.	Sansonetti PJ, Arondel J, Cantey JR, Prévost MC, Huerre M. Infection of rabbit Peyer’s patches by Shigella flexneri: Effect of adhesive or invasive bacterial phenotypes on follicle-associated epithelium. Infect Immun. 1996;64: 2752–2764. Available: http://www.ncbi.nlm.nih.gov/pubmed/8698505

9.	Eberl G, Lochner M. The development of intestinal lymphoid tissues at the interface of self and microbiota. Mucosal Immunol. 2009;2: 478–485. doi:10.1038/mi.2009.114

10.	Agace WW, McCoy KD. Regionalized Development and Maintenance of the Intestinal Adaptive Immune Landscape. Immunity. 2017;46: 532–548. doi:10.1016/j.immuni.2017.04.004

11.	Microanatomical dissection of human intestinal T-cell immunity reveals site-specific changes in gut-associated lymphoid tissues over life. - PubMed - NCBI [Internet]. [cited 27 Jan 2020]. Available: https://www.ncbi.nlm.nih.gov/pubmed/30523311

12.	Jacob E, Baker SJ, Swaminathan SP. “M” cells in the follicle-associated epithelium of the human colon. Histopathology. 1987;11: 941–52. doi:10.1111/j.1365-2559.1987.tb01900.x

13.	Fujimura Y, Hosobe M, Kihara T. Ultrastructural study of M cells from colonic lymphoid nodules obtained by colonoscopic biopsy. Dig Dis Sci. 1992;37: 1089–1098. doi:10.1007/BF01300292

14.	Ohno H. Intestinal M cells. J Biochem. 2015;159: 151–60. doi:10.1093/jb/mvv121

15.	d’Hauteville H, Sansonetti PJ. Phosphorylation of IcsA by cAMP-dependent protein kinase and its effect on intracellular spread of Shigella flexneri. Mol Microbiol. 1992;6: 833–841.

16.	Allaoui A, Sansonetti PJ, Parsot C. MxiD, an outer membrane protein necessary for the secretion of the Shigella flexneri lpa invasins. Mol Microbiol. 1993;7: 59–68. doi:10.1111/j.1365-2958.1993.tb01097.x

17.	Kocks C, Gouin E, Tabouret M, Berche P, Ohayon P, Cossart P. L . monocytogenes-Induced Actin Assembly Requires the actA Gene Product , a Surface Protein. Cell. 1992;68: 521–531.

18.	Shetron-Rama LM, Marquis H, Bouwer HGA, Freitag NE. Intracellular induction of Listeria monocytogenes actA expression. Infect Immun. 2002;70: 1087–1096. doi:10.1128/IAI.70.3.1087-1096.2002

19.	Dailey ME, Marrs GS, Kurpius D. Maintaining live cells and tissue slices in the imaging setup. Cold Spring Harb Protoc. 2011;6: 373–380. doi:10.1101/pdb.top105

20.	Bhagat V, Becker ML. Degradable Adhesives for Surgery and Tissue Engineering. Biomacromolecules. 2017;18: 3009–3039. doi:10.1021/acs.biomac.7b00969

21.	Lo D, Tynan W, Dickerson J, Scharf M, Cooper J, Byrne D, et al. Cell culture modeling of specialized tissue: Identification of genes expressed specifically by follicle-associated epithelium of Peyer’s patch by expression profiling of Caco-2/Raji co-cultures. Int Immunol. 2004;16: 91–99. doi:10.1093/intimm/dxh011
